# Mutation in LEMD3 (Man1) Associated with Osteopoikilosis and Late-Onset Generalized Morphea: A New Buschke-Ollendorf Syndrome Variant

**DOI:** 10.1155/2016/2483041

**Published:** 2016-06-13

**Authors:** Benjamin Korman, Jun Wei, Anne Laumann, Polly Ferguson, John Varga

**Affiliations:** ^1^Division of Rheumatology, Northwestern University Feinberg School of Medicine, Chicago, IL, USA; ^2^Department of Dermatology, Northwestern University Feinberg School of Medicine, Chicago, IL, USA; ^3^Department of Pediatrics, University of Iowa Carver College of Medicine, Iowa City, IA, USA

## Abstract

*Introduction*. Buschke-Ollendorf syndrome (BOS) is an uncommon syndrome characterized by osteopoikilosis and other bone abnormalities, accompanied by skin lesions, most frequently connective tissue nevi. BOS is caused by mutations in the* LEMD3* gene, which encodes the inner nuclear membrane protein Man1. We describe a unique case of osteopoikilosis associated with late-onset localized scleroderma and familial* LEMD3* mutations.* Case Report*. A 72-year-old woman presented with adult-onset diffuse morphea and bullous skin lesions. Evaluation revealed multiple hyperostotic lesions (osteopoikilosis) suggestive of BOS. DNA sequencing identified a previously undescribed nonsense mutation (Trp621X) in the* LEMD3* gene encoding Man1. Two additional family members were found to have osteopoikilosis and carry the same* LEMD3* mutation.* Conclusions and Relevance*. We report a unique familial* LEMD3* mutation in an individual with osteopoikilosis and late-onset morphea. We propose that this constellation represents a novel syndromic variant of BOS.

## 1. Introduction

Osteopoikilosis is a rare autosomal dominant skeletal dysplasia characterized by multiple hyperostotic lesions. The bone lesions are generally symmetric but distributed irregularly and are typically detected as incidental radiographic findings [1]. Osteopoikilosis can be an isolated skeletal abnormality or may occur in association with diverse cutaneous manifestations as a component of Buschke-Ollendorf syndrome (BOS) (OMIM166700) [2, 3]. The cutaneous manifestations of BOS, commonly manifesting in childhood, include connective tissue nevi and less frequently elastomas, collagenomas, and dermatofibrosis lenticularis (also called hypertrophic scar disseminata) [4–6]. The genetic basis for BOS was identified in 2004 by genome-wide linkage studies. These studies uncovered a mutation in* LEMD3* (LEM domain containing 3) gene [2]. The* LEMD3* gene encodes the 60 kD inner nuclear membrane protein Man1. Mutations in* LEMD3* are also linked to skeletal abnormalities other than BOS. These include isolated (nonsyndromic) osteopoikilosis [2] and melorheostosis, a hyperostotic anomaly characterized by radiolucent “dripping wax” appearance in the cortex of long bones [7, 8]. Of note, melorheostosis itself may be an isolated radiological finding or occur in association with abnormalities in adjacent soft tissue, including linear scleroderma [9–12].

Morphea is a localized form of scleroderma characterized by skin induration in localized areas. Morphea has the highest incidence in childhood and young adults. Late-onset morphea is considerably less common. In contrast to systemic sclerosis, morphea is confined to the skin and is not associated with extracutaneous manifestations. The spectrum of morphea disorders includes linear scleroderma, plaque morphea, and diffuse morphea, which in rare cases may be extensive (pansclerotic morphea). Morphea lesions commonly occur on the extremities and the face and less frequently on the trunk. The etiology of morphea is unknown and its pathogenesis remains poorly understood.

Transforming growth factor-beta (TGF-*β*) is a multifunctional cytokine implicated in fibrosis in multiple organs [13]. The profibrotic responses elicited by TGF-*β* involve both Smad-dependent canonical, as well as Smad-independent noncanonical intracellular signaling pathways [14, 15]. Alterations in TGF-*β* expression or function and in its downstream signaling mediators are implicated in the pathogenesis of localized scleroderma and systemic sclerosis [16]. Man1, the protein encoded by* LEMD3,* is intricately linked to TGF-*β* biology and has complex effects on modulating TGF-*β* responses. On one hand, Man1 interacts directly with TGF-*β* superfamily ligands, including bone morphogenic proteins (BMPs) and activin [17]. On the other hand, Man1 binds, via its C-terminal domain, directly to Smad [17, 18]. Importantly, Man1 negatively regulates Smad-mediated TGF-*β* signaling in a variety of cell types [2, 17–23]. Despite these recent molecular insights, the full spectrum of* LEMD3* mutations and their impact on TGF-*β* biology and their functional role in the phenotypic expression of BOS remain poorly understood.

Genetic variants of* LEMD3* have been associated with distinct clinical phenotypes in addition to BOS. These include isolated osteopoikilosis and melorheostosis [1, 2, 8, 24–30]. We propose that this case represents a novel variant of BOS.

## 2. Case Report

**Figure 1:**
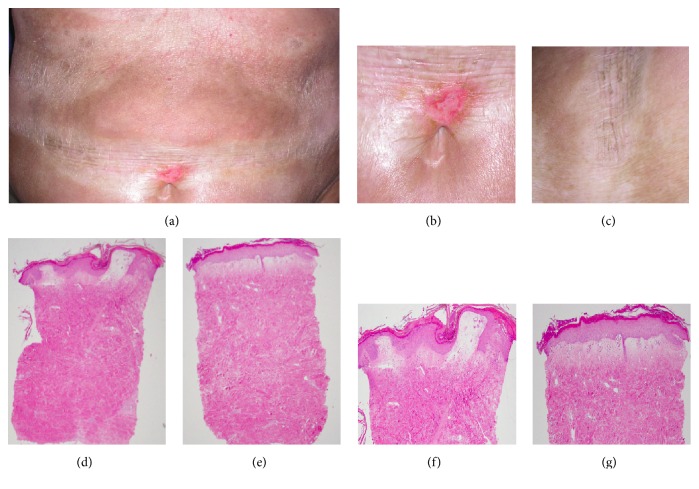
Clinical and dermatopathological findings in a patient carrying a novel LEMD3/Man1 (Trp620X) mutation. (a–c) Morphea lesions. (a) Skin lesions involving abdomen. Note indurated periumbilical skin. (b) Close-up highlighting ruptured periumbilical bulla. (c) Indurated skin on chest. (d–g) Histopathology from lesional skin. (d, f) Abdominal skin, H&E, 20x (d) and 50x (f). (e, g) Chest skin, H&E stain 20x (e) and 50x (g).

A previously healthy 72-year-old Caucasian woman presented with six months' progressive skin tightening and discoloration affecting her arms, shoulders, chest, and lower legs. Subsequently, painful erythematous patches appeared on her back, breasts, and belt line. She had no family history of scleroderma or other autoimmune disease. Physical examination demonstrated firmly indurated and hyperpigmented lesions on the arms, shoulders, chest, belt line, and lower legs and scaly erythematous and partially bullous patches over both breasts (Figures 1(a)–1(c)). She had no sclerodactyly, nailfold microvascular abnormalities or other manifestations of systemic sclerosis, and serologic tests for antinuclear, anti-Scl-70, and anti-centromere antibodies were negative. Radiographs of the hands, feet, and knees revealed numerous well-demarcated bone densities (osteopoikilosis) bilaterally (Figure 3). Based on the presence of osteopoikilosis and skin lesions, the diagnosis of BOS was made, and genomic DNA sequencing was undertaken (see below). Further investigation identified three family members (school-aged nieces and nephews on the paternal side) who had asymptomatic osteopoikilosis, but no skin lesions. Treatment of the index case included psoralens and ultraviolet light A, oral calcitriol hydroxychloroquine, and mycophenolate mofetil, as well as topical calcipotriene, betamethasone dipropropionate, and pimecrolimus. She showed slow partial resolution of skin lesions. Subsequent course was complicated by recurrent episodes of hemorrhagic olecranon bursitis and hemorrhagic bullae over the chest, abdomen, and back.

### 2.1. Cutaneous Histopathology

**Figure 2:**
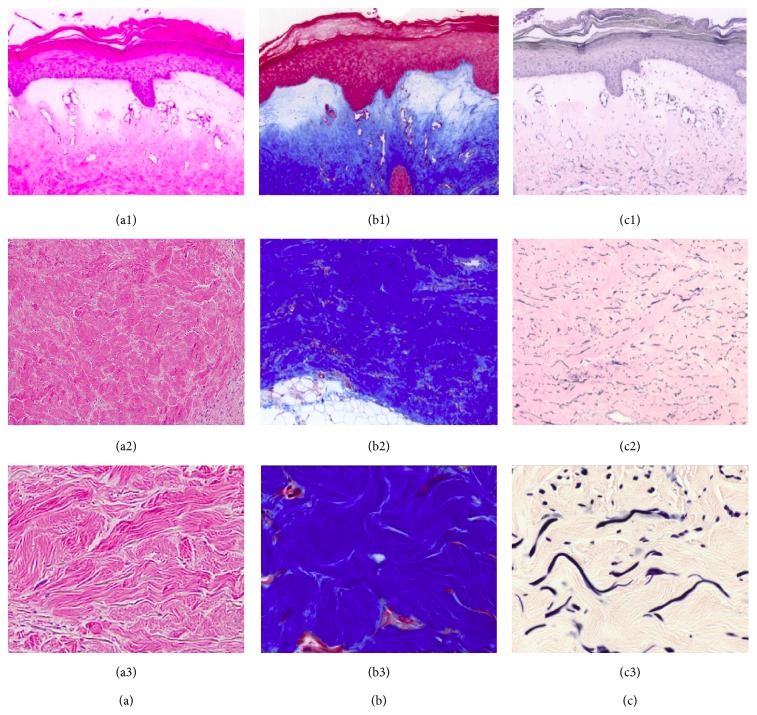
Histochemistry of lesional skin from patient with LEMD3 Trp620X mutation. (a) H&E stain. (b) Masson's trichrome staining. (c) Elastin staining. (a1), (b1), and (c1) represent 100x magnification of the epidermis, (a2), (b2), and (c2) represent 100x magnification of the dermis, and (a3), (b3), and (c3) show 630x magnification of the dermis. Note increase in both collagen and elastin deposition and irregular collagen fibrils.

**Figure 3:**
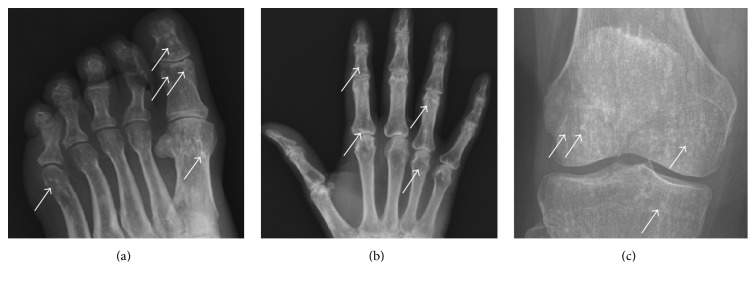
Osteopoikilosis. Plain radiographs. (a) Foot. (b) Hand. (c) Knee. Note multiple small (1–5 mm) sclerotic periarticular lesions consistent with bony islands of osteopoikolosis (marked by arrows).

A punch biopsy of lesional skin yielded square-shaped tissue with fibrosis and a cellular infiltrate (Figures 1(d)–1(g)). The upper dermis showed bullous changes including edema and dilated vessels consistent with lichen sclerosus et atrophicus. Masson's trichrome and elastin stains revealed dense dermal collagen deposition and increased elastic fiber accumulation (Figure 2).

### 2.2. DNA Sequencing

**Figure 4:**
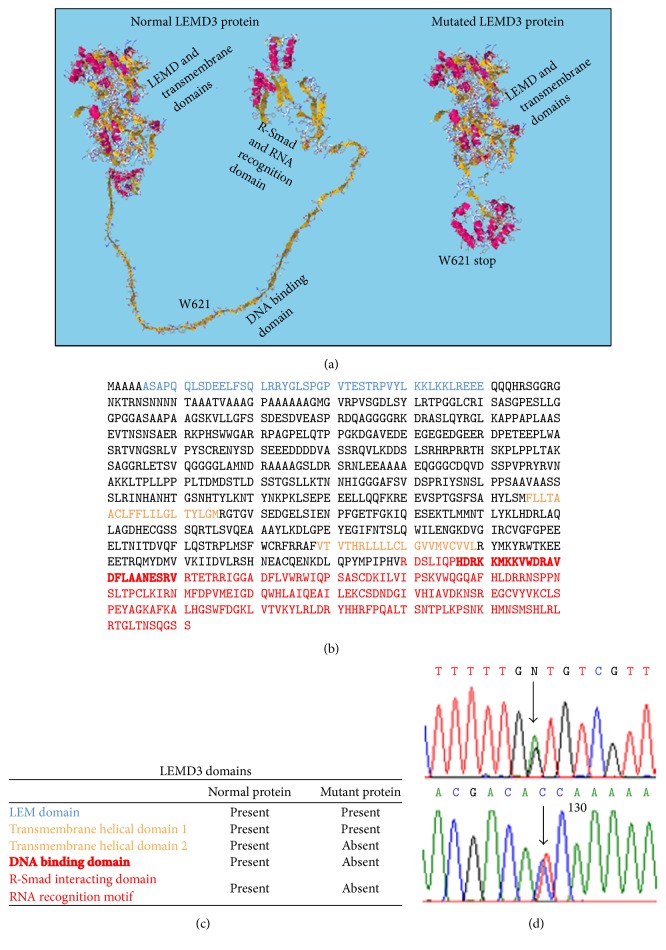
Characterization of novel LEMD3 mutation. (a) 3D predicted conformation of native and mutated p.Trp620X Man1 protein (EsyPred3d modeling software) [31]. Note deletion of the DNA-binding and R-Smad recognition domains. (b) Amino acid sequence of Man1; letters represent amino acids as defined by IUPAC. The Trp620X codon is indicated. (c) List of functional domains and presence of domains in normal and mutated Man1 protein. (d) DNA sequence of* LEMD3*, highlighting the novel c.1863G > A mutation.

Index case DNA was extracted from peripheral blood using a commercial kit (Sigma, St. Louis, MO). Sanger sequencing of the entire* LEMD3* gene identified a heterozygous nonsense mutation c.1863G > A which results in a change at amino acid 621 that converts a tryptophan residue to a stop codon (p.Trp621X). This nucleotide change is predicted to truncate Man1 at amino acid 621, resulting in deletion of the second transmembrane helical domain and DNA-binding and Smad-interacting domains [31] (Figure 4). The mutant gene product is predicted to lack the Smad-binding domain of Man1 required for antagonizing TGF-*β* signaling. This* LEMD3* mutation was not present in the exome variant server database (http://evs.gs.washington.edu/EVS/) representing 13,000 control alleles [including 8,600 alleles from individuals of European descent] or in the 1000 Genomes Project database (http://www.1000genomes.org/).

## 3. Literature Survey and Discussion

First described in 1928, BOS is an uncommon familial syndrome characterized by osteopoikilosis associated with skin manifestations [32, 33]. In children with BOS, osteopoikilosis has been reported to be accompanied by fibrotic skin lesions, including linear scleroderma, part of the morphea spectrum disorders [5, 6, 34–36]. We are unaware of a previous description of late-onset generalized morphea associated with osteopoikilosis.

The present case might represent the coexistence of two distinct disorders affecting the skin and bone. We consider this unlikely however. As osteopoikilosis has an estimated prevalence of 2/100,000 and morphea of 0.02–0.04/100,000 [37], the extreme rarity of these two conditions makes their occurrence in the same individual by chance highly unlikely. A favored alternative explanation is that late-onset generalized morphea associated with osteopoikilosis seen in the present case is in fact syndromic and represents a novel BOS variant that falls within the phenotypic continuum linked with* LEMD3* mutations.

Previous studies have led to identification of* LEMD3* as the gene that is mutated in BOS [2]. In addition, different* LEMD3* mutations have also been linked with nonsyndromic familial forms of both osteopoikilosis and melorheostosis [2]. In order to review current knowledge of BOS and its cutaneous manifestations, a PubMed survey using the search terms “BOS”, “Ollendorf Buschke”, “Buschke-Ollendorf”, “osteopoikilosis”, “melorheostosis”, “LEMD3”, and “Man1” was undertaken (Table 1). Over 30 reported cases with* LEMD3* loss-of-function mutations linked with these phenotypes were identified [1, 2, 8, 24–28, 30, 38, 39] (Table 1). Cutaneous manifestations include connective tissue nevi, fibrous nodular lesions (collagenomas or elastomas), and linear scleroderma [26].

A review of over 100 published cases of BOS showed that connective tissue nevi (dermatofibrosis lenticularis disseminata) were the most frequent cutaneous manifestation. The diagnosis of BOS was characteristically made before the age of 16. A survey of cases of* LEMD3*-*associated skin and bony lesions* revealed 28 cases of melorheostosis associated with linear scleroderma, typically affecting skin adjacent to the bone lesions, with a majority of these individuals developing linear (localized) scleroderma in childhood (Table 2). However, melorheostosis frequently occurs in the absence of* LEMD3* mutations [8], and thus far none of the* LEMD3* mutation-proven cases of melorheostosis (Table 1) have coincided with linear scleroderma. One report of osteopoikilosis associated with scleroderma described a patient with sclerodactyly and Raynaud phenomenon, suggesting coexistent systemic sclerosis and isolated osteopoikilosis rather than syndromic BOS [6].

*LEMD3* mutations show variable penetrance. There is extreme variability in the associated phenotypes, even among individuals harboring identical mutations [2]. Given such a high degree of heterogeneity and incomplete penetrance, the causal role of any particular* LEMD3* mutation in a specific phenotype is difficult to discern. Although the TGF-*β*/Smad signaling pathway plays a pivotal role in both skin and bone homeostasis, it remains unclear how Man1-Smad interactions are affected by the BOS mutations, and whether they contribute to clinical features. While the novel* LEMD3* mutation described in this report is predicted to alter the C-terminal domain of Man1 required for R-Smad interactions [23], our functional studies failed to demonstrate consistent alterations in TGF-*β*/Smad signaling in the BOS skin fibroblasts.

The coexistence of morphea and lichen sclerosus et atrophicus (LSA) changes is also of note. While this combination has been previously reported as a cause of bullous changes [40–43] in morphea, the association is relatively common in adults. A recent retrospective study confirmed the coexistence of these two entities in 26 of 91 (28.5%) of adult morphea patients compared to only 1 of 381 children with morphea [44]. Bullous LSA changes are primarily inflammatory [45] and some have suggested that LSA may represent subepithelial morphea in this context [46]. Therefore, whether the LSA changes are related to the LEMD3 mutation or are simply part of the morphea phenotype is unclear.

## 4. Summary

In summary, we describe a case of osteopoikilosis associated with late-onset generalized morphea and associated LSA changes in an elderly individual carrying a previously undescribed familial mutation in* LEMD3*. We propose that in this case morphea and osteopoikilosis are linked, representing a novel BOS variant that is on the continuum of* LEMD3*-associated skin and bone manifestations. In light of the known involvement of Man1 in modulating canonical TGF-*β* signaling, we hypothesize that the skin and bone abnormalities associated with* LEMD3* mutations might be related to altered TGF-*β* signaling. Future studies will characterize the functional consequences of* LEMD3* mutations and their role in the clinical manifestations of the syndrome. Given the diverse phenotypes associated with such mutations and poorly understood mechanisms of how Man1 protein changes contribute to the phenotypic manifestations of BOS, such studies may reveal new roles for this diverse molecule in mesenchymal cell biology.

## Figures and Tables

**Table 1:** Previously reported *LEMD3* mutations.

Point mutations	94X	Buschke-Ollendorff syndrome [30]

(Missense/nonsense)	457C > T	Osteopoikilosis [2]
	620X	Buschke-Ollendorff syndrome (present study)
	641X	Buschke-Ollendorff syndrome [4]
	1323C > A	Osteopoikilosis [28]
	1609C > T	Osteopoikilosis and Melorheostosis [2]
	1801G > T	Osteopoikilosis [8]
	1873C > T	Melorheostosis [39]
	1913T > A	Melorheostosis [8]
	2032C > T	Osteopoikilosis [25]
	2203C > T	Buschke-Ollendorff syndrome [1]
	2564G > A	Buschke-Ollendorff syndrome [29]

Insertions/deletions/duplications/indels	332_333 insTC	Buschke-Ollendorff syndrome [28]
	830 dupA	Melorheostosis [8]
	1033–1035 delGGGinsC	Osteopoikilosis [2]
	1185 dupT	Osteopoikilosis [2]
	1914 dupA	Buschke-Ollendorff syndrome [8]
	1941 +5delG	Osteopoikilosis [2]
	2154 dupA	Osteopoikilosis [2]
	Entire gene deletion	Osteopoikilosis [2]

None		Buschke-Ollendorff syndrome [47]

Splicing	IVS1 ds +1 G-A	Collagenoma [26]
	IVS12 ds +1 G-A	Buschke-Ollendorff syndrome [48]

**Table 2:** Cases of scleroderma-spectrum disease and *LEMD3*-type bony lesions.

Study (1st author)	Juvenile-onset linear scleroderma	Adult-onset linear scleroderma	Systemic sclerosis	Generalized morphea	Melorheostosis	Osteopoikilosis
Thompson [49]	x				x	
Maroteaux [9]	x				x	
Muller [10]	x				x	
Pascaud-Ged [50]	x				x	
Moreno Alvarez [51]	x				x	
Saghafi [7]	x				x	
Soffa [52]	x				x	
Takeda [53]	x				x	
Nakajima [54]	x				x	
Miyachi [55]	x				x	
Siegel [56]		x			x	
Birtane [57]		x			x	
Endo [58]		x			x	
Shivanand [12]		x			x	
Weissmann [6]			x			x
*Present case*				x		x

x: presence of feature in case report.
